# Case report: Success of allogeneic hematopoietic stem cell transplantation for refractory systemic-onset juvenile idiopathic arthritis

**DOI:** 10.3389/fmed.2023.1275927

**Published:** 2023-10-16

**Authors:** Camille Beaufils, Catherine Proulx, Annaliesse Blincoe, Pierre Teira, Henrique Bittencourt, Sonia Cellot, Michel Duval, Marie-Paule Morin, Jean Jacques De Bruycker, Julie Couture, Kathryn Samaan, Hélène Decaluwe, Niina Kleiber, Ramy El-Jalbout, Fabien Touzot, Elie Haddad, Julie Barsalou

**Affiliations:** ^1^Division of Pediatric Rheumatology and Immunology, CHU Sainte-Justine, Montreal, QC, Canada; ^2^Department of Pediatrics, CHU Sainte-Justine, Université de Montréal, Montreal, QC, Canada; ^3^Division of Pediatric Hemato-Oncology, CHU Sainte-Justine, Montreal, QC, Canada; ^4^Division of Pediatric Rheumatology, Immunology and Allergy, CHU Sainte-Justine, Montreal, QC, Canada; ^5^Department of Radiology, CHU Sainte-Justine, Université de Montréal, Montreal, QC, Canada

**Keywords:** systemic-onset juvenile idiopathic arthritis, juvenile idiopathic arthritis, pediatric rheumatic disease, allogeneic hematopoietic stem cell transplantation, bone marrow transplantation

## Abstract

**Objectives:**

This study reports cases of systemic-onset juvenile idiopathic arthritis (sJIA) who underwent allogeneic hematopoietic stem cell transplantation (allo-HSCT) at our center and reviews published outcomes of allo-HSCT in sJIA.

**Methods:**

We present a case report of two patients with sJIA who underwent allo-HSCT at a tertiary pediatric hospital. Each patient’s disease course and allo-HSCT protocol/outcome are described. Outcomes of published cases of allo-HSCT in sJIA were compared to our experience.

**Results:**

Two patients with sJIA had allo-HSCT. Both failed multiple lines of disease-modifying anti-rheumatic drugs and experienced severe disease/treatment-related complications. Despite post-HSCT complications, both recovered without sequelae. Five years post-HSCT, patient 1 is in complete remission (CR) and is off medications. Patient 2 was in CR until 11 months post-HSCT after which he developed three disease flares. At 4 years post-HSCT he is currently in CR on Adalimumab monotherapy. Engraftment was excellent with a donor chimerism of 100% for patient 1 and 93% for patient 2. In the literature, the outcome of allo-HSCT is reported in 13 sJIA patients. When merging those with our 2 patients, 1/15 patients died and 13/14 achieved CR, of which 12 are off medications (median [range] follow-up: 2.2 [0.2–7.0] years). Extended follow-up data on 11 of the 13 reported sJIA patients showed that an additional 3 patients flared at 3, 4, and 10 years post-HSCT.

**Conclusion:**

We report two patients with severe/refractory sJIA who underwent successful allo-HSCT and achieved CR. Allo-HSCT is a potential curative option for severe/refractory sJIA. It should be considered only after failure of conventional sJIA treatments and when an HLA-matched donor is available in order to lower transplant-related mortality. The outcomes of reported sJIA patients who received allo-HSCT are encouraging but long-term follow-up data are needed to better characterized the risk–benefit ratio of this procedure.

## Introduction

Biologic therapies have improved the outcome of systemic-onset juvenile idiopathic arthritis (sJIA) (1). However, some patients with severe/refractory disease represent a challenge. Those patients not only face disease complications but also treatment-related toxicities.

Autologous hematopoietic stem cell transplantation has been reported in patients with autoimmune disease (AID) since 1997, but allogeneic-HSCT (allo-HSCT) has been described more recently (2). Although allo-HSCT introduces greater risks of complications, it is more likely to induce long-term remission off immunosuppression (IS) medication (3, 4). There is little data on allo-HSCT in sJIA. We report our successful experience with allo-HSCT in two children with severe/refractory sJIA. The evolution of these two patients was compared to cases published in the literature.

## Patients and methods

All sJIA patients who received an allo-HSCT at our tertiary care center were included in this case report. Patients’ demographics, disease course, allo-HSCT protocol, and outcomes were collected retrospectively. The CHU Sainte-Justine Research Ethics Board (REB) does not require REB reviews for case reports. Written patient/parental informed consent was obtained for publication.

A PubMed search was performed to retrieve cases of allo-HSCT in sJIA published in English/French scientific journals. The following keywords were used: systemic-onset juvenile idiopathic arthritis, juvenile idiopathic arthritis, juvenile rheumatoid arthritis, hematopoietic stem cell transplantation, bone marrow transplantation, and transplantation. Titles/abstracts were screened (CB, JuB) for relevance, and reports in which data of sJIA could be extracted were selected. Disease features and HSCT procedure/outcomes were summarized and compared to the course of our patients.

## Cases description

Two patients with sJIA underwent allo-HSCT at our center.

**Figure 1:**
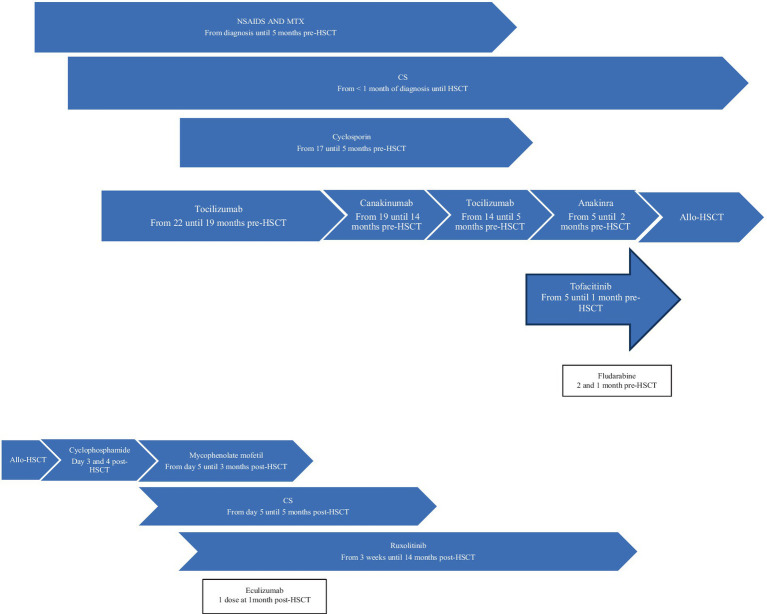
Patient 1 treatment timeline. Allo-HSCT, allogeneic hematopoietic stem cell transplantation; CS, corticosteroids; MTX, methotrexate; NSAIDS, Non-steroidal anti-inflammatory drugs.

**Patient 1** was diagnosed with sJIA at 3 years old (Table 1). She was assessed at our center 1.4 years after her initial diagnosis and remained on moderate/high doses of corticosteroids (CS). The decision to proceed to allo-HSCT was based on ongoing disease activity refractory to eight lines of therapy as well as severe disease/treatment-related complications (Table 1 and Figure 1). She developed numerous thoracolumbar vertebral compression fractures and extensive (T2-sacrum) epidural lipomatosis leading to severe spinal stenosis/spinal cord compression. Three months before HSCT, decompressive laminectomy/surgical lipomatosis excision was performed. In an effort to wean CS to the lowest possible dose, a course of fludarabine was administered 2 and 1 month prior to transplant. Despite this, her disease remained clinically/biologically active. Levels of interleukin-18 (IL-18) or other cytokines were not performed. Genetic panels for familial hemophagocytic lymphohistiocytosis (fHLH) and autoinflammatory disorders were performed prior to HSCT and were negative.

**Table 1:** Patient characteristics and evolution post allo-HSCT.

	Patient 1	Patient 2
Gender	F	M
Disease manifestations	Quotidian fever; Rash; Polyarthritis; MAS	Quotidian fever; Rash, Polyarthritis; Splenomegaly
Refractory disease manifestations	Systemic symptoms; Polyarthritis; MAS	Systemic symptoms; Polyarthritis
Previous treatments	NSAID; CS; Methotrexate; Tocilizumab; Anakinra; Canakinumab; Cyclosporin; Tofacitinib	NSAID; CS; Methotrexate; Tocilizumab; Canakinumab; Infliximab; Tofacitinib; Siltuximab
Disease and treatment-related toxicities	Severe Cushingoid habitus; Obstructive sleep apnea; Severe osteoporosis with multiple vertebral fractures; Spinal epidural lipomatosis/spinal stenosis causing spinal cord compression; Hypertensive cardiomyopathy; Severe hepatic steatosis; Small stature/growth delay	Severe Cushingoid habitus; Joint erosions; C1-C2 instability; Osteopenia with peroneal fracture; HTN; Severe hepatic steatosis; Small stature/growth delay; Cataracts
Age at HSCT, years	5.4	7.5
Donor type	mURD	MRD
Source	Peripheral blood stem cell	Bone marrow
Conditioning regimen	Fludarabine 180 mg/m^2^; Busulfan (AUC =12,711 μM.min); Alemtuzumab 5 mg/kg	Fludarabine180 mg/m^2^; Busulfan (AUC =12,807 μM.min); ATG 7.5 mg/kg
Post-HSCT IS	Cyclophosphamide; MMF; Ruxolitinib; CS; Eculizumab (1 dose)^a^	Cyclosporin; MMF; CS; Rituximab; Tofacitinib; Adalimumab
Post-HSCT complications^b^	Inflammatory flares; Severe HTN with PRES; Grade II acute cutaneous and GI GVHD; Grade III mucositis; Multifactorial transient renal failure (including possible TMA); Transient hepatitis; HSV reactivation; Suspected BOOP vs. BOS; Negative coagulase staphylococcus bacteriemia; Bacterial lower urinary tract infection	Grade I mucositis; Rhinovirus pneumonia; Cyclosporin renal toxicity; BK virus cystitis; EBV reactivation
Time to stop IS post-HSCT, months	14	10; 25; ongoing^c^
Time to stop CS post-HSCT, months	5^d^	0.8^e^
Chimerism (years post-HSCT)	100% donor on whole blood (5.2)	93% donor on whole blood (4.0)
Disease outcome (years post-HSCT)	CR off IS (5.6)	sJIA relapse (0.9; 2.3; 2.9); CR on Adalimumab (4.0)
Beneficial effects	Resolution of Cushingoid habitus; Improved BMI (37 to 24); Improved growth velocity (<3rd to 3rd percentile); Resolved obstructive sleep apnea; No recurrence of spinal cord compression; Normalized blood pressure; Resolution of hepatic steatosis.	Improved Cushingoid habitus; Improved BMI (37 to 28); Improved growth velocity (<3rd to 3rd–25th percentile); Normalized bone density on DEXA scan; Normalized blood pressure; Improved liver enzymes.

ATG, Antithymocyte globulin; BMI, Body mass index; BOOP, Bronchiolitis obliterans with organizing pneumonia; BOS, Bronchiolitis obliterans syndrome; CR, Complete remission; CS, corticosteroids; DEXA, Dual-energy x-ray absorptiometry; EBV, Epstein–Barr virus; F, Female; GI, Gastro-intestinal; GVHD, Graft-versus-host disease; HSCT, Hematopoietic stem cell transplantation; HSV, Herpes simplex virus; HTN, Hypertension; IS, Immunosuppression; LVH, Left ventricular hypertrophy; M, Male; MAS, Macrophage activation syndrome; MMF, Mycophenolate mofetil; mURD, Matched unrelated donor; MRD, Matched related donor; NSAID, Non-steroidal anti-inflammatory drugs; PRES, Posterior reversible encephalopathy syndrome; sJIA, Systemic-onset juvenile idiopathic arthritis; TMA, thrombotic microangiopathy.

a Given for possible thrombotic microangiopathy.

b Both patients recovered without sequelae from all complications listed.

c IS stopped at 10 months post-HSCT but patient flared; second trial to stop IS was performed 25 months post-HSCT but patient flared; IS is ongoing since the second disease flare.

d Remained on physiologic doses of CS from month 6–18 post-HSCT.

e Remained on physiologic doses of CS from day + 11 to month 12 post-HSCT, received one pulse (30 mg/kg) of methylprednisolone at 10 months post-HSCT.

Allo-HSCT was performed 1.8 years after her initial diagnosis (Table 1). Within the first 24 h, patient 1 developed life-threatening symptoms, including fever, elevated inflammatory markers, and hypotension requiring hemodynamic support. In the following 5 months, she experienced multiple episodes of polyarthralgia and erythematous rash, initially associated with fever and elevated inflammatory markers, that were very similar to her sJIA flares. Methylprednisolone was added to her IS regimen. She improved and tolerated CS weaning. The underlying etiology of these inflammatory episodes was never confirmed. She had numerous non-inflammatory-related complications from which she recovered without sequelae. Corticosteroids and all other IS medications were stopped at 5 and 14 months post-HSCT, respectively. At 5.6 years post-allo-HSCT, she remains in CR off IS medications with full donor chimerism.

**Figure 2:**
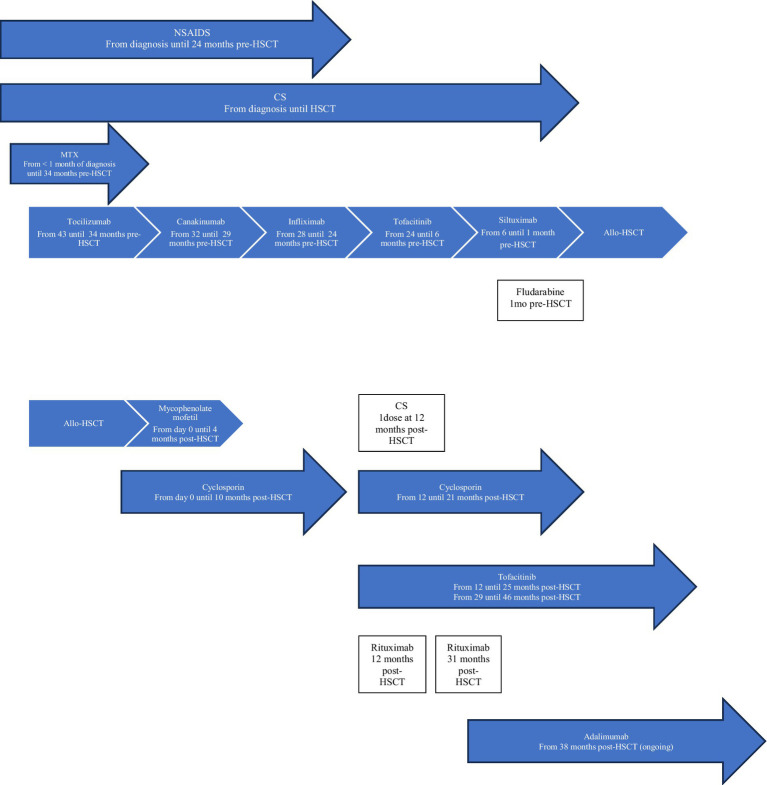
Patient 2 treatment timeline. Allo-HSCT, allogeneic hematopoietic stem cell transplantation; CS, corticosteroids; MTX, methotrexate; NSAIDS, Non-steroidal anti-inflammatory drugs.

**Patient 2** was diagnosed with sJIA at the age of 4 years old (Table 1). He was on moderate/high doses of CS since his diagnosis (3.8 years) (Figure 2). Severe/refractory disease and disease/treatment-related complications prompted consideration for allo-HSCT. Patient 2 had developed severe steatohepatitis. Extensive polyarthritis had resulted in significant restrictions, requiring the use of a wheelchair. The level of IL-18 was measured once 8 months prior to HSCT during a phase of active systemic and joint disease and was elevated (18,095 pg./mL). Genetic testing for fHLH did not reveal explanatory variants. As for patient 1, a course of fludarabine was started 1 month pre-HSCT, and this enabled a decrease in CS pre-transplant.

At the time of allo-HSCT, patient 2 was 7.5 years old with a disease duration of 3.8 years (Table 1). No major complications occurred during HSCT. He experienced episodic polyarthralgia in the first 3 months following HSCT. All IS medications were stopped 10 months post-transplant. One month later, he experienced a first disease flare (quotidian fever, polyarthritis, elevated inflammatory markers). This flare was concomitant with a decrease in whole blood (WB) donor chimerism (99% at day+24, and 88% at the time of flare) as well as an increase in serum EBV DNA (5,623 log10 copies). He received a single pulse of methylprednisolone (30 mg/kg), was restarted on cyclosporin/tofacitinib, and had a course of rituximab. He responded rapidly to this treatment and re-achieved CR. His WB donor chimerism increased to 95%. Cyclosporin/tofacitinib was again stopped at 2.1 years post-HSCT. He had a second disease flare (polyarthritis without systemic symptoms) at 2.3 years post-HSCT. Whole blood, CD3+ T cells, and neutrophil donor chimerisms were at 93%, 86%, and 98%, respectively. This second flare was also associated with an increase in serum EBV DNA (35,481 log10 copies). Tofacitinib was reintroduced, and a second course of rituximab was given. Again, he showed major improvement over the following months. He developed a SARS-CoV2 infection 2.8 years post-HSCT, and 1 month later he presented with a third disease flare (polyarthritis without systemic symptoms). Whole blood donor chimerism at that time was stable (94%). Serum EBV DNA was undetectable. Despite an increase in the dose of tofacitinib, his polyarthritis remained, and he was thus started on adalimumab (3.2 years post-HSCT). He showed gradual improvement and attained CR on medication 3.8 years post-HSCT. Tofacitinib was stopped. At the most recent follow-up appointment (4 years post-HSCT), his WB donor chimerism was 93%, and he remains in CR on adalimumab monotherapy. More extensive genetic testing is ongoing for monogenic etiology.

## Literature review

Our PubMed search retrieved 147 references. After excluding 44 duplicates, 103 abstracts were reviewed, and 13 additional references were included. Among 116 screened abstracts, 107 were excluded as they were not reporting on allo-HSCT in sJIA. In six papers it was unclear whether reported cases had sJIA and/or what type (auto vs. allo) of HSCT was performed and/or no information on the HSCT outcome was provided (3, 5–9). We were left with three manuscripts in which outcomes of allo-HSCT in 13 sJIA were reported (Table 2) (10–12). Patients were transplanted after a median disease duration of 3.3 years and had failed 1–7 lines of DMARDS (excluding non-steroidal anti-inflammatory drugs and CS). The donor type/source, conditioning, and graft-versus-host disease (GVHD) prophylaxis are found in Table 2. There was one death due to pulmonary hemorrhage in the context of GVHD/systemic fungal infection. Among 12 surviving patients, 7 had complete donor chimerism. Complete remission off IS was achieved in 11/12 (median [range] follow-up of 2.1 [0.8–7.0] years). The median (range) time to stop IS in 10/11 (data unavailable for one patient) was 0.6 (0.5–2.2) years. A total of 2 out of 12 surviving patients had disease flares: 1 patient had a fever, skin rash, and arthralgia with raised inflammatory markers and was treated with CS at 22 months post-HSCT. He then went into CR off IS. The second patient had arthritis responsive to CS 2 months post-HSCT and a macrophage activation syndrome (MAS)-like episode responsive to IS 6 months post-HSCT. He then went into partial remission (PR) on IS. Extended follow-up information on 11 sJIA patients originally published by Silva et al. revealed that three additional patients had a disease flare at 3, 4, and 10 years (13). All three patients remain on IS medication following disease relapse. With this updated data, the number of sJIA patients in CR off IS is 8/12.

**Table 2:** Published procedures and outcomes of allo-HSCT in sJIA.

Number of patients (references)	13 (10–12)
Age at HSCT, years	6.2 (2.7–16.8)
Disease duration at HSCT, years	3.3 (1.3–15.3)
Donor type (*N*)	MUD (5); mMUD (2); MSD (5); HAPLO (1)
Conditioning regimen (*N*)	F/M/A (8); F/T/A (3) B/F/A/RTX (1); F/M/THIO/A (1)
GVHD prophylaxis	MMF/CsA^a^ CYC/CsA/MMF (1); Tac/MMF (1)
Donor chimerism, % (*N*)^b^	100^c^ (7) 89^d^ (1); 88^d^ (1); 67^d^ (1); 55^d^ (1) <10^d^ (1)
Time to stop IS, years [*N*]^e^	0.6 (0.5–2.2) [10] Ongoing (2)
Follow-up post-HSCT, years	2.0 (0.2–7.0)
Remission (*N*)	CR (11)^f^ PR (1)
Flare (*N*)	2
Death (*N*)	*N* = 1

Data is shown as median (range) unless otherwise specified. A, Alemtuzumab; B, Busulfan; CR, Complete remission; CS, Corticosteroids; CsA, Cyclosporin; CYC, Cyclophosphamide; F, Fludarabine; GVHD, Graft-versus-host disease; HAPLO, Haploidentical donor; HSCT, Hematopoietic stem cell transplantation; IS, Immunosuppression; M, Melphalan; MAS, Macrophage activation syndrome; MMF, Mycophenolate mofetil; mMUD, Mismatched unrelated donor; MSD, Matched sibling donor; MUD, Matched unrelated donor; N/A, Not available; PR, Partial remission; RIC, Reduced intensity conditioning; RTX, Rituximab; T, Treosulfan; Tac, Tacrolimus; THIO, Thiotepa.

a The paper by Silva et al. mentions that “most patients received MMF and cyclosporin” but individual treatment was not specified.

b Data available for N = 12 patients and timing of measurement not specified for most patients.

c Performed on peripheral blood/whole blood for patients reported by Silva et al.

d Performed on CD3.

e Data unavailable for one patient.

f One patient is in CR of his sJIA symptoms but remains with lung disease which is described as significantly improved.

## Discussion

We report two patients with sJIA who underwent allo-HSCT due to severe/refractory disease. Now at 5.6 years following HSCT, patient 1 remains in CR off IS medication, is free of short−/medium-term complications from the procedure, and reports a dramatically improved quality of life. Patient 2 had three disease flares post-HSCT but responded to treatment each time. At 4 years post-HSCT, he is in CR on adalimumab monotherapy. The procedure is still regarded as a success considering the patient’s pre-HSCT state with poly-IS requirements. Interestingly, both patients experienced intermittent polyarthralgias without arthritis in the first few months following allo-HSCT, which is associated with significant inflammatory surges for patient 1. It is possible that an intrinsic defect in dendritic cells was driving both patients’ diseases; therefore, a certain degree of immune reconstitution had to take place before improvement.

When comparing our two patients with the cases summarized in Table 2, the median (range) time to stopping IS post-HSCT, 0.6 (0.5–2.2) years, was shorter than in our patients (1.2 years and ongoing). When we include the two patients presented in this report with those already published (*N* = 15), it is encouraging to note that transplantation-related mortality is low (*N* = 1/15, 7%) (10–12). Initial observation showed that after a median (range) of 2.2 (0.2–7.0) years following HSCT, 12/14 patients are in CR off IS. Three patients had disease flares post-HSCT: two re-achieved CR (one on/one off IS) and one achieved PR on medication. When including the updated follow-up data from Silva et al., the number of patients in CR off IS lowers to 9/14 (13). The latter reflects the importance of having longer-term follow-up data. This will help to better understand the place of allo-HSCT in the treatment armamentarium for severe and refractory sJIA cases.

Reports of autologous HSCT (auto-HSCT) in JIA have also shown interesting results. Among 22 children with JIA (of whom 18 had sJIA) who underwent auto-HSCT and were followed for a median period of 80 (range 52–104) months, 18 children survived (14). A total of 2 children died of MAS within days and months of the procedure, respectively. The other two deaths occurred between year 1 and 2 post-HSCT (complications of viral infections while being on immunosuppressive medications to treat disease relapse). Among the 18 surviving children, 8 were in CR off IS medication and 7 were considered partial responders (50%–70% improvement). Failure of auto-HSCT was documented in 3 patients. Morbidities associated with the procedure were mainly due to infectious complications occurring early post-auto-HSCT. Another group summarized the outcome of published cases of auto-HSCT in JIA (which included the cohort discussed above) and reported that among 57 JIA patients, 31 were in CR off IS, 6 were in PR, 13 had disease relapse, and 9 died (13). No prospective trials comparing auto vs. allo-HSCT in sJIA exist. Further studies are needed to explore which HSCT modality offers the best benefit/risk ratio in the sJIA population.

Reports of allo-HSCT in patients with various AID have shown less optimistic results. A retrospective study of the European Society for Blood and Marrow Transplantation registry reported outcomes of 128 patients with hematologic (*N* = 49) and non-hematologic (*N* = 79) AID who underwent allo-HSCT between 1997 and 2014 (5). JIA was the indication for HSCT in 13/128 patients, which included the 11 sJIA patients reported by Silva et al. The median (range) age at transplantation of the entire cohort was 12.7 (0.2–62.2) years. Among 128 patients, the relapse incidence was 20% at 5 years. The overall survival was 70.2% at 5 years. Morbidity/mortality were higher in this cohort of AID. Many factors may explain the differences in outcomes, including different underlying disease, donor type/source, longer time since HSCT, and inclusion of adults.

When considering allo-HSCT for the treatment of sJIA, several factors should be taken into account. The first one is the timing of transplantations. The main advantage of allo-HSCT is its potential to be curative and allow cessation of IS. Allo-HSCT should not be viewed as a “last resort” option and delayed until the accumulation of irreversible and severe disease/treatment complications, which render the procedure obsolete and increase transplantation-related morbidities and mortality. On the other hand, allo-HSCT should not be considered too early as more aggressive therapy with upfront biologics or combination therapy may alter the disease course even in severe cases. The second important factor is the choice of donor. The disease could result from single or additive genetic defects with incomplete penetrance. In this case, using a sibling donor would expose the recipient to a risk of disease recurrence. This issue needs to be discussed with patients/families. In addition, screening for known monogenic mimickers of sJIA should be performed prior to HSCT. With advances in the field of HSCT, comparable outcomes between the matched related donor and matched unrelated donor have been reported in the oncology literature (15–18). Fewer data exist on the AID population (5). A third point to consider is the intensity of the conditioning regimen since it can influence the allo-HSCT’s outcome. The optimal conditioning maximizes donor engraftment with the lowest degree of toxicity. We used fludarabine/busulfan reduced-toxicity conditioning (RTC) regimen. The downside of RTC is the risk of graft rejection and mixed chimerism that could lead to disease persistence after HSCT. Of note is that none of our patients encountered graft rejection. It remains unclear whether the existence of mixed chimerism contributed to the sJIA flare in patient 2. It is possible that for some patients, a higher level of donor chimerism is required for disease cure. The minimally effective level of engraftment and the lineage specificity required to cure patients with sJIA remains unknown. A recent review on levels of donor engraftments obtained to achieve disease remission in diverse non-malignant conditions displayed wide ranges of *effective* donor chimerism, ranging from 10% to 100% (19). Finally, it is noteworthy that even with full chimerism, patient 1 presented with sJIA-like manifestations in the first 5 months following HSCT, suggesting the persistence of tissue-resident recipient cells despite the conditioning regimen that could be ultimately cleared by a hypothetical graft-versus-autoinflammatory disease effect. Interleukin-18 has been shown to correlate with sJIA disease course (20). It is unknown if and how it can help to identify potential candidates for transplant and assistance during post-HSCT follow-up period, for example, to help distinguish between primary disease recurrence vs. post-HSCT complications (engraftment syndrome, infections, etc.). Other biomarkers might be of interest to this specific population.

Limitations of our report are the small number of patients described and the relatively short follow-up period post allo-HSCT. However, few sJIA patients treated with allo-HSCT have been reported to this date, and existing cohorts often comprise merged AID diagnoses of adults and children, which makes comparison of outcomes difficult.

## Patient perspective

Patient 1 is highly satisfied with her outcome following allo-HSCT. Being in CR and off all IS has improved her quality of life. Patient 2 has verbalized being disappointed in not reaching CR off IS. Being in disease remission, especially being off CS, was an important milestone for him and his family.

In conclusion, allo-HSCT was successful in achieving CR in two patients with severe/refractory sJIA. Allo-HSCT should only be considered in carefully selected patients, after failure of conventional sJIA treatments, and when an HLA-matched donor is available in order to lower transplant-related mortality. Prospective studies addressing the long-term efficacy/safety of allo-HSCT in sJIA are urgently needed to better define the role of this potential curative therapeutic option of sJIA.

## Data availability statement

The data underlying this article will be shared on reasonable request to the corresponding author.

## Ethics statement

Ethical approval was not required for the study involving humans in accordance with the local legislation and institutional requirements. Written informed consent to participate in this study was not required from the participants or the participants’ legal guardians/next of kin in accordance with the national legislation and the institutional requirements. Written informed consent was obtained from the participants’ legal guardians for publication of this case report.

## Author contributions

CB: Conceptualization, Data curation, Formal analysis, Investigation, Methodology, Writing – original draft, Writing – review & editing. CP: Conceptualization, Data curation, Formal analysis, Investigation, Methodology, Writing – original draft, Writing – review & editing. AB: Formal analysis, Investigation, Writing – review & editing. PT: Formal analysis, Investigation, Writing – review & editing. HB: Formal analysis, Investigation, Writing – review & editing. SC: Formal analysis, Investigation, Writing – review & editing. MD: Formal analysis, Investigation, Writing – review & editing. M-PM: Formal analysis, Investigation, Writing – review & editing. JeB: Formal analysis, Investigation, Writing – review & editing. JC: Formal analysis, Investigation, Writing – review & editing. KS: Formal analysis, Investigation, Writing – review & editing. HD: Formal analysis, Investigation, Writing – review & editing. NK: Formal analysis, Investigation, Writing – review & editing. RE-J: Formal analysis, Investigation, Writing – review & editing. FT: Formal analysis, Investigation, Writing – review & editing, Data curation, Methodology. EH: Formal analysis, Investigation, Methodology, Writing – review & editing, Conceptualization. JuB: Conceptualization, Formal analysis, Investigation, Methodology, Writing – review & editing, Data curation, Project administration, Supervision, Validation, Writing – original draft.
